# Spontaneous Epithelial-Mesenchymal Transition and Resistance to HER-2-Targeted Therapies in HER-2-Positive Luminal Breast Cancer

**DOI:** 10.1371/journal.pone.0071987

**Published:** 2013-08-26

**Authors:** David Lesniak, Siham Sabri, Yaoxian Xu, Kathryn Graham, Pravin Bhatnagar, Mavanur Suresh, Bassam Abdulkarim

**Affiliations:** 1 Department of Experimental Oncology, Cross Cancer Institute and University of Alberta, Edmonton, Alberta, Canada; 2 Department of Radiation Oncology, Research Institute of McGill University Health Center, McGill University, Montréal, Quebec, Canada; 3 Faculty of Pharmacy and Pharmaceutical Sciences, University of Alberta, Edmonton, Alberta, Canada; University of South Alabama, United States of America

## Abstract

Resistance to trastuzumab, a rationally designed HER-2-targeting antibody, remains a major hurdle in the management of HER-2-positive breast cancer. Preclinical studies suggest the mechanisms of trastuzumab resistance are numerous. Unfortunately, the majority of these studies are based around HER-2-positive (HER-2+) luminal cell lines. The role of epithelial to mesenchymal transition (EMT), a genetic program that confers a basal phenotype, may represent a novel mechanism of escape for HER-2+ luminal cells from trastuzumab treatment. Here we investigated this possibility using a model of clonal selection in HER-2+ luminal breast cancer cells. Following a random isolation and expansion of “colony clusters” from SKBR-3 cell lines, several colony clusters underwent a spontaneous EMT in-vitro. In addition to expression of conventional EMT markers, all mesenchymal colony clusters displayed a predominant CD44+/CD24- phenotype with decreased HER-2 expression and elevated levels of a β1-integrin isoform with a high degree of N-glycosylation. Treatment with a β1-integrin function-blocking antibody, AIIB2, preferentially decreased the N-glycosylated form of β1-integrin, impaired mammosphere formation and restored epithelial phenotype in mesenchymal colony clusters. Using this model we provide the first clear evidence that resistance to trastuzumab (and lapatinib) can occur spontaneously as HER-2+ cells shift from a luminal to a basal/mesenchymal phenotype following EMT. While the major determinant of trastuzumab resistance in mesenchymal colony clusters is likely the down regulation of the HER-2 protein, our evidence suggests that multiple factors may contribute, including expression of N-glycosylated β1-integrin.

## Introduction

In recent years, research emphasizing the cellular hierarchy and architecture within the mammary gland has greatly enhanced our understanding of breast cancer (BC). Breast ducts have two main epithelial cell types: basal and luminal cells are bounded by a fibrous basement membrane and embedded in a growth mediating stroma rich for soluble factors [1]. Accumulating evidence points to a cellular hierarchy in the mammary gland, whereby stem cells (SC) residing in the basal layer give rise to basal and luminal cell types [2]. Together basal, luminal and SC provide the overall genetic background for four of five intrinsic subtypes, including: claudin-low (SC enriched), basal, and two luminal subtypes. The fifth intrinsic subtype is called HER-2-positive (HER-2+), and results from overexpression or amplification of the HER-2 oncogene [3].

Approximately, 15–25% of invasive breast tumors are HER-2+, a designation associated with aggressive clinical disease and poor overall prognosis [4]. As a subgroup, HER-2+ tumors can demonstrate luminal and/or basal BC features. Interestingly, a recent clinical study found that HER-2+ tumors with an under-ridding basal phenotype have a worse overall prognosis than basal tumors alone [5]. Nonetheless, the standard medical care for HER-2+ BC remains trastuzumab, a HER-2 directed antibody. In HER-2+ BC, trastuzumab in combination with conventional chemotherapy extends the disease and progression-free survival [6], [7]. Unfortunately, rates of intrinsic resistance to trastuzumab-based treatment range from 30–50%. For those who initially respond, acquired resistance to trastuzumab typically occurs in less than one year [8], [9].

Based on preclinical studies, potential mechanisms of trastuzumab resistance (TZR) include dysregulated mitogenic signaling from alternative signaling receptors or loss of tumor suppressor PTEN [9]–[11]. Interestingly, the HER-2+ cell lines used in most preclinical studies are of predominantly luminal phenotype [12]. This may significantly impact our current understanding of TZR, especially relating to recent “cancer SC” (CSC)-based models. For instance, HER-2+ luminal cell lines are typically enriched for a distinct CSC population defined by high aldehyde dehydrogenase (ALDH) activity. These CSC express the highest HER-2 levels and remain sensitive to trastuzumab treatment [13], [14].

Interestingly, JIMT-1, the first commercial cell line established from a HER-2+ patient with intrinsic resistance to trastuzumab [15], is naturally enriched for a CD44+, CD24 negative (CD44+/CD24-) CSC phenotype characteristic for basal/mesenchymal cells [16]. JIMT-1 also expresses high levels of β1-integrin, a basal adhesion molecule responsible for mediating most cell-matrix interactions with the breast basement membrane [17]. In breast development, β1-integrin is essential for proper SC function and tissue morphogenesis [18]. In BC, β1-integrin is implicated in nearly every aspect of tumor development and progression, including drug resistance and the induction of epithelial to mesenchymal transition (EMT) [19]–[22]. Interestingly, we recently identified β1-integrin as the strongest prognostic indicator of TZR in a cohort of HER-2+ metastatic BC. Additionally, when siRNA was used to knockdown β1-integrin in JIMT-1 cells, they failed to survive in culture [23].

One potential link between trastuzumab sensitive HER-2+ luminal cells and trastuzumab resistant basal/mesenchymal cells is EMT. EMT is an embryonic transcription network characterized by a CD44+/CD24- CSC phenotype and increased malignant behavior [24]. In a recent study, Oliveras-Ferraros et al, demonstrated the importance of TWIST and SLUG in maintaining the EMT phenotype of JIMT-1 cells. Accordingly, depletion of SLUG in JIMT-1 diminished the percentage of cells with a CD44+/CD24- CSC phenotype, and sensitized JIMT-1 to trastuzumab treatment [25]. A previous study by the same authors showed that spontaneous enrichment of the CD44+/CD24- CSC phenotype in JIMT-1 coincided with a global decrease in HER-2 expression [16]. Together, these results demonstrate a role for EMT in maintaining the intrinsic trastuzumab resistant phenotype of HER-2+ basal/mesenchymal cell line, JIMT-1. However, the role of EMT in trastuzumab sensitive, HER-2+/luminal cells remains to be investigated.

Here we investigated the role of EMT in TZR using luminal HER-2+ SKBR-3 cells transfected with an empty expression vector or a vector containing β1-integrin. When distinct colony clusters were isolated simultaneously from luminal SKBR-3 using multiple cloning rings, several underwent a spontaneous EMT in culture. Unlike conventional “single cell” cloning techniques, which yield very few expandable cell populations [26], all colony clusters of SKBR-3 survived the expansion phase in culture. Characterization of this “de-novo” EMT-phenotype in SKBR-3 revealed decreased HER-2 levels and expression of a heavily N-glycosylated variant of β1-integrin, associated malignant traits in BC cells [27]. Here we present original evidence that TZR can result from the spontaneous conversion of HER-2+ cells to a CD44+/CD24-/HER-2-low phenotype through EMT. Furthermore, our results suggest a function link between EMT, N-glycosylated β1-integrin and basal CSC in HER-2+ BC.

## Materials and Methods

### Cell culture and reagents

SKBR-3 (American Type Culture Collection) and JIMT-1 (Deutsche Sammlung von Mikroorganismen und Zellkulturen GmbH) were cultured in DMEM with 10% fetal bovine serum and 1% antibiotic/antimycotic (Invitrogen) in a 5% CO_2_-humidified incubator at 37°C (standard conditions). Trastuzumab (Genentech) was purchased from the pharmacy at the Cross Cancer Institute. Lapatinib Ditosylate (Biovision) was solubilized in DMSO. Rat-IgG1 and Human-IgG1 (Jackson Immuno Research Laboratories) served as control antibodies for AIIB2 and trastuzumab, respectively. N-Glycosidase-F (PGNase-F) was used as recommended by the manufacturer (New England Biolabs).

### Clone isolation

SKBR-3 clones were selected from cells stably transfected with empty vector (SKBR-3/EV) or β1-integrin (SKBR-3/β1) as described previously [23]. Briefly, cells were seeded at low density onto 10cm plates (100,000 cells total). Using multiple 10mm cloning rings, “colony clusters” were isolated simultaneously from a single plate of cells after 10–12 days growth (standard conditions). Please refer to Methods S1 for cloning schematic and legend detailing the procedure.

### Western blotting

Lysate (30 µg) was processed for immunoblotting as previously described [23]. Primary antibodies are listed. Cell signaling technology: HER-2 (2242), phospho-Akt (193H12), Vimentin (5G3F10), Cytokeratin 8/18 (C51), TWIST-1 (4119), alpha5-integrin (4705), alpha6-integrin (3750), phospho-FAK (pY397), N-Cadherin (32). Others: β1-integrin (clone18, BD Transduction), total Akt (H136, Santa Cruz), total FAK (4.47, Upstate), and β-actin (AC-15, Sigma-Aldrich).

### Mammosphere formation

Single cell suspensions (10,000 cells/ml) were plated in ultra-low attachment 24 well plates (1ml/well) (3473, Costar). **Media composition:** DMEM/F-12, 0.75% methylcellulose, 1X B27 supplement, 10 ng/ml EGF and FGF-2, 5µg/ml insulin, 0.5 μg/ml hydrocortisone. The number of mammospheres (> 80 μm in diameter) was quantified after 10–14 days.

### Image capture and morphological assessment

A Seniscam camera attached to a Zeiss Axiovert 200M microscope with a Zeiss Plan-NEOFLUAR 5x/0.15 or 10x/0.3 lens was used unless otherwise stated. MetaMorph v7.0 was used to export images (TIFF). Adobe Photoshop CS4 was used to process images (levels and cropping). Error bars  = 100 μm. Cell Profiler (www.cellprofiler.org) [28] was used for morphology analysis. Briefly, a pipeline to mask and outline cells was generated from randomly selected images. Cell masks were assessed for eccentricity (cell resemblance to a circle), generating a value between 0 (circle) to 1 (line) for each cell.

### Gene expression profiling

Agilent Whole-Genome Arrays were analyzed with Genespring-GX-7.3 software. Sample preparation was performed as previously described [29]. Gene expression profiles from post-EMT (passage >5) cultures of SK-EV-C4, SK-β1-C1, and passage-matched cultures of SKBR-3/EV, SKBR-3/β1, were compared. Features selected had at least a 2X difference in at least one cell-cell comparison (one-way ANOVA, *P*-value >0.05). Graphs presented as fold change relative to gene expression in SKBR-3/EV.

### Hierarchical clustering and pathway analysis

Expression profiles from SKBR-3/EV and SK-β1-C1 were compared. A differential gene list was generated to include genes differing by at least 1.5X, with a t-test *P*-value of >0.02 (unpaired, Bonferroni corrected). A total of 1940 entities met these criteria, including β1-integrin and HER-2. Canonical Pathways Analysis (Ingenuity Systems, www.ingenuity.com) was performed using a Fisher's exact test. Hierarchical clustering was performed using Euclidean distance metric and centroid linkage and displayed as a heat Map (Genespring).

### AIIB2 production and purification

See Methods S1. Briefly, β1-integrin positive, AIIB2 hybridoma cells detected by indirect ELISA were grown in hyperflask culture vessels with 10 gas permeable layers (Corning). Rat-monoclonal antibody, AIIB2, was purified from the supernatant. **AIIB2 treatment:** Following overnight attachment, cells in standard conditions were treated for 72 h with AIIB2 or rat-IgG (monolayer culture). For mammosphere culture, growth media was supplemented with AIIB2 or IgG at indicated concentrations. Mammospheres were assessed as described above.

### Trastuzumab and lapatinib sensitivity

Cells were seeded into 96-well microtiter plates (1,500/well in 100 μL) in standard conditions. Media was treated with trastuzumab, human IgG1, lapatinib or vehicle at indicated concentrations. After 72 h, cell proliferation was assessed as previously described [23]. Results presented relative to control condition.

### Conditioned media

An equal number of SKBR-3/EV or SK-β1-C1 cells (500,000 cells/60 mm dish) were allowed to grow overnight in standard conditions. Media was collected the following day, spun at 500 g for 3 min to remove cellular debris and dead cells. Supernatant was mixed 1∶1 with fresh standard media prior to use.

### Statistics

Where applicable, results are presented as mean plus standard deviation (SD) from at least 3 independent experiments performed in triplicate or quadruplicate. Significance assessed using two-tailed student t-tests with a *P*-value <0.05 considered to be significant.

## Results

### De-novo SKBR-3 phenotype is mediated by spontaneous EMT, and results in conversion to a HER-2 negative basal/mesenchymal phenotype

When we cultured SKBR-3 cells as clones, phenotypic heterogeneity was apparent between colonies. Comparatively, no heterogeneity was observed between colonies in basal/mesenchymal cell line, JIMT-1 (data not shown). We used multiple cloning rings simultaneously on a single dish of cultured cells to isolate distinct clusters of SKBR-3 colonies (“colony clusters”) transfected with either an empty expression vector or one containing basal marker, β1-integrin. We also isolated colony clusters from trastuzumab resistant JIMT-1 cells known to have elevated levels of endogenous β1-integrin **(Fig. S1)**.

**Figure 1:**
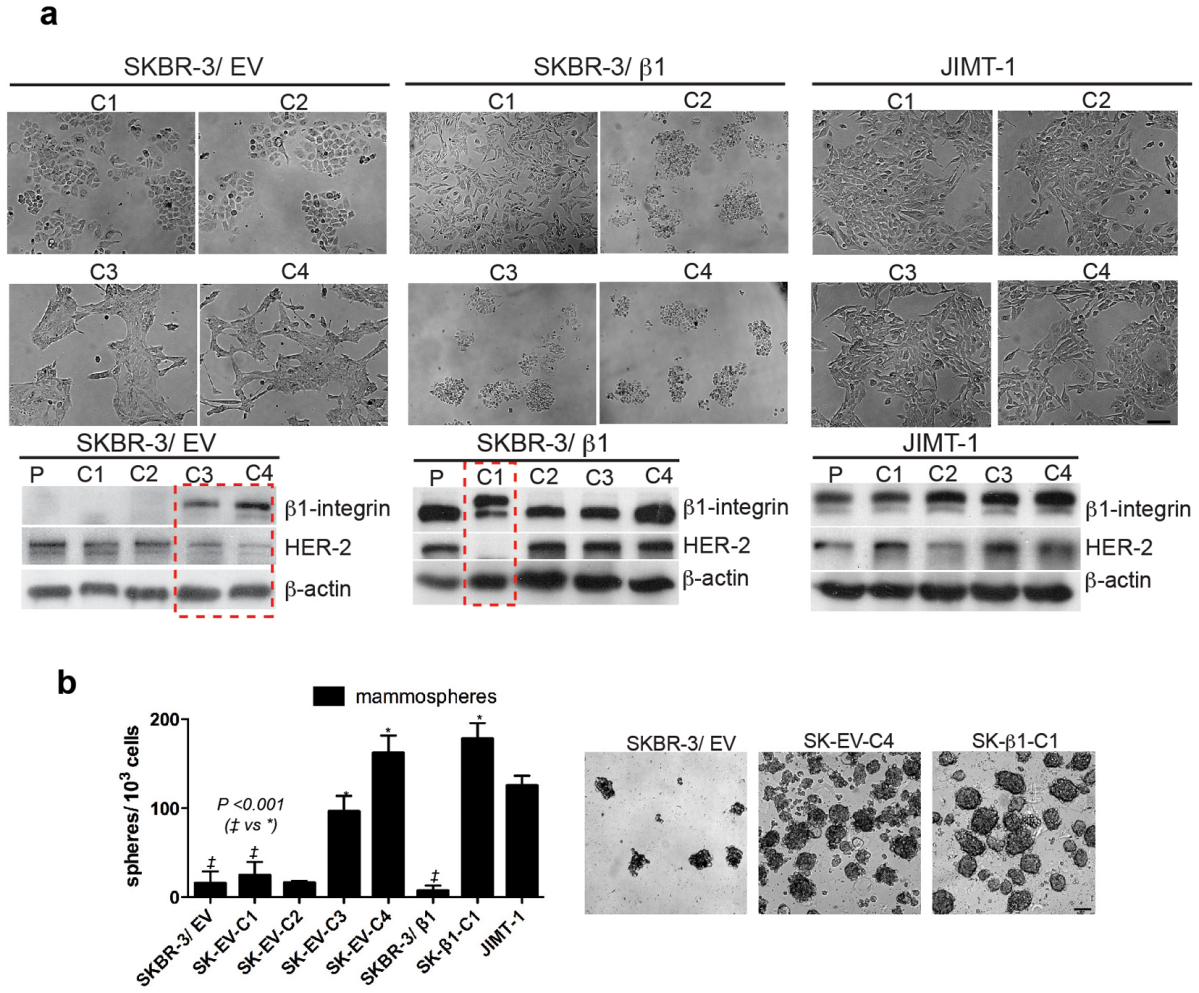
Spontaneous EMT in SKBR-3 colony clusters is characterized by mature β1-integrin isoform and decreased HER-2. [**a**] Images and western blotting for HER-2 and β1-integrin confirm a de-novo phenotype occurred in 2/4 colony clusters (abbreviated “C”), isolated from SKBR-3/EV (SK-EV-C3, EV-C4), and 1/4 colony cluster isolated from SKBR-3/β1 (SK-β1-C1) (dashed boxes). No de-novo phenotype was observed in any JIMT-1 colony cluster. [**b**] Mammosphere formation assessed and imaged following day 10 showed increased mammosphere formation in SK-EV-C3, EV-C4 and SK-β1-C1 compared to SKBR-3/EV and SKBR-3/β1.

Unexpectedly, a spontaneous phenotype transition occurred in several SKBR-3 colony clusters shortly following isolation. Compared to the typical round (luminal) morphology of SKBR-3 cells, 2/4 colony clusters (50%) from SKBR-3/EV (SK-EV-C3, EV-C4) and 1/4 colony cluster (25%) from SKBR-3/β1 (SK-β1-C1) acquired spindle (mesenchymal) morphology. Interestingly, all colony clusters isolated from JIMT-1 retained a mesenchymal morphology following colony selection. Notably, western blotting revealed that SKBR-3 colony clusters with a de-novo phenotype expressed low levels of HER-2 and high levels of a β1-integrin variant with decreased electrophoretic motility, a similar expression pattern found in JIMT-1 cells (**Fig. 1a**).

Next, we used mammosphere formation as a functional measure of the de-novo SKBR-3 phenotype. Our results showed that cell lines retaining a round morphology (SKBR-3/EV, SK-EV-C1, EV-C2, and SKBR-3/β1) formed relatively scarce populations of mammospheres compared to cell lines that acquired spindle morphology (SK-EV-C3, EV-C4 and SK-β1-C1). The latter forming a large number of rapidly expanding mammospohere populations (*P*-value <0.001) (**Fig. 1b**). By serially passaging SK-β1-C1 mammospheres, we confirmed an increased capacity for self-renewal in these cells (**Fig. S2**). By comparison, SKBR-3/β1 cells that expressed only low molecular weight β1-integrin formed only small primary mammospheres and no second-passage mammospheres (data not shown).

**Figure 2:**
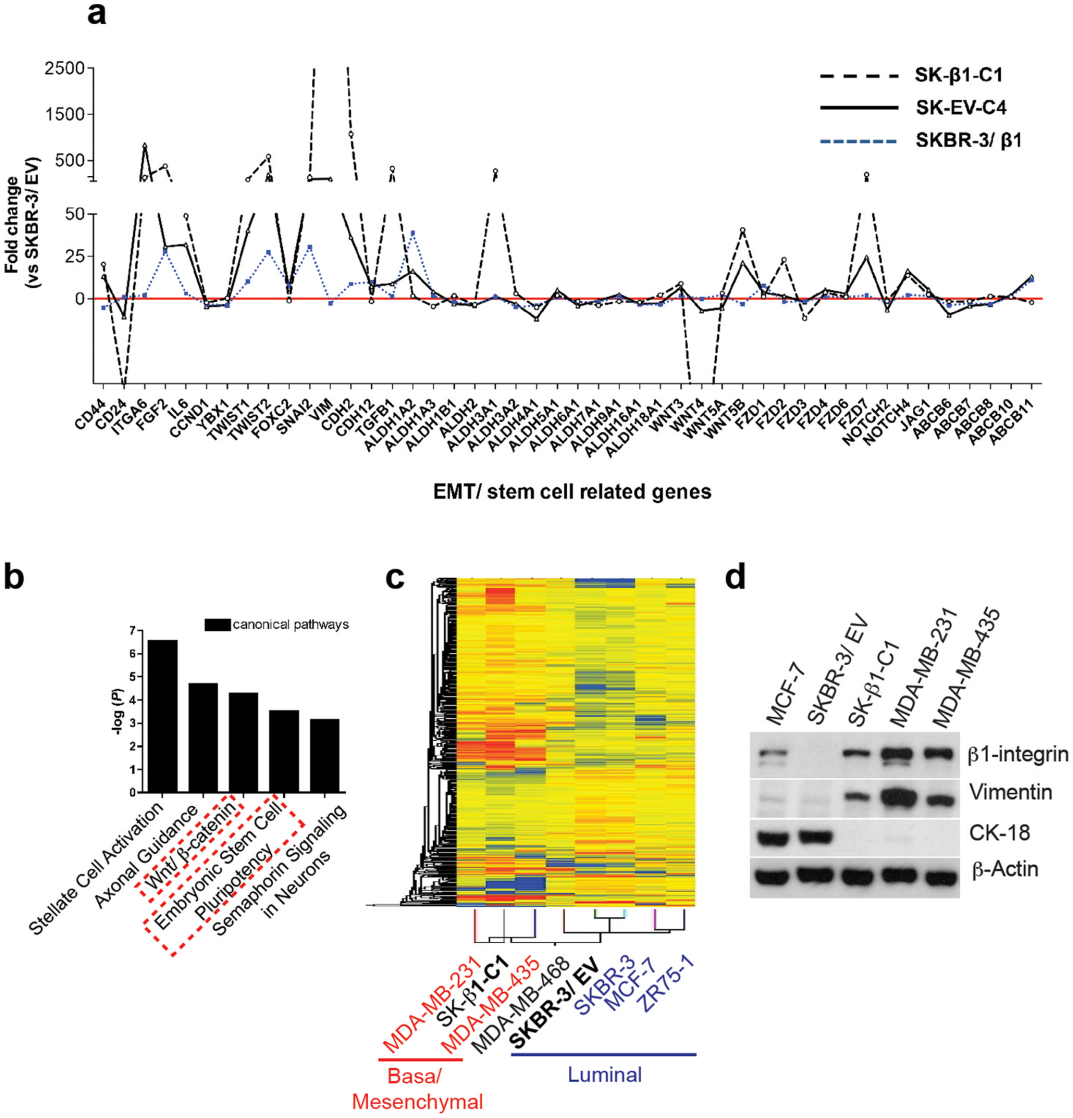
EMT results in the conversion from a luminal to a basal/mesenchymal phenotype including expression of CSC related gene networks. [**a**] Relative gene expression differences for major EMT/CSC markers, between luminal (SKBR-3/EV, SKBR-3/β1), and mesenchymal (SK-EV-C4, SK-β1-C1) colony clusters. [**b**] Canonical pathway analysis based on differentially expressed genes between SKBR-3/EV and SK-β1-C1 (n = 1940 genes). Stem cell related gene networks are upregulated in SK-β1-C1 (dashed boxes). [**c**] Unsupervised hierarchical clustering of SKBR-3/EV and SK-β1-C1 based on the 1940 gene set. Basal/mesenchymal cell lines (MDA-MB-231, MDA-MB-435) cluster with SK-β1-C1 while luminal, HER-2 negative cell lines (MCF-7, ZR75-1) cluster with SKBR-3/EV. [**d**] Western blotting for expression of β1-integrin, vimentin, and cytokeratin-18 (CK-18).

To determine the molecular phenotype and identify heterogeneity among different SKBR-3 colony clusters, we used gene expression profiling. Gene expression data from luminal SKBR-3/EV and SKBR-3/β1 cells, together with mesenchymal colony clusters, SK-EV-C4 and SK-β1-C1, was compared and then normalized to SKBR-3/EV for analysis. Data was analyzed for differences in HER receptors, integrins, and major SC markers (P-value cutoff  = 0.05). In terms of HER receptors, EGFR levels remained similar between all cell lines. Alternatively, HER-2 and HER-3 levels were significantly decreased in mesenchymal colony clusters, SK-EV-C4 and SK- β1-C1 (**Fig. S3**). Interestingly SC related alpha6-integrin was upregulated in mesenchymal colony clusters but not in luminal SKBR-3/β1 cells (**Fig. 2a**).

Next, we assessed several EMT/SC markers including, ALDH family members, MDR (multi drug resistant) transporters, CD44, CD24, IL-6, YBX1, NOTCH and WNT pathway proteins. We found a significant up-regulation of CD44 together with down-regulation of CD24 in SKBR-3 colony clusters following EMT. In addition, IL-6, WNT ligand Frizzled 7 (FZD 7), WNT5B, and NOTCH4 were all upregulated in mesenchymal colony clusters compared to luminal cell lines. No heterogeneity in MDR transporter gene transcription was noted between SKBR-3 cell lines, and gene expression analysis for ALDH family members revealed minimal alterations to this protein family. Notably, ALDH1A2 was highly upregulated in luminal SKBR-3 cells transfected with low molecular weight β1-integrin (SKBR-3/β1). Other differences in ALDH proteins included down-regulation of ALDH4A1 exclusively in SK-EV-C4 and up-regulation of ALDH7A1 in SK-β1-C1. Classical-EMT markers: vimentin (VIM), N-cadherin (CDH2, CDH12), TWIST-1, SLUG (SNAI2), and fibronectin (FN) were upregulated in de-novo mesenchymal colony clusters (**Fig. 2a**). Using flow cytometry we confirmed the CD44+/CD24- protein pattern in SK-β1-C1 cells (**Fig. S4**). In terms of β1-integrin, we again performed flow cytometry, confirming increased expression levels in SK-β1-C1 (data not shown). Additionally, we used western blotting to confirm gene expression differences of several integrin family members, including SC-related, alpha6-integrin, increased in SK-β1-C1 following EMT (**Fig. S4**).

Pathway analysis was conducted using a set of differentially regulated genes (n = 1940) between luminal SKBR-3/EV and mesenchymal SK-β1-C1. Notably, SC-associated pathways, including: Wnt/beta-catenin and embryonic SC pluripotency, were among the top five canonical signaling networks upregulated in SK-β1-C1 (**Fig. 2b**). Furthermore, the top upregulated genes in SK-β1-C1 cells included two ECM proteins with β1-integrin binding specificity, collagen-1A2 and lamanin-alpha-4, as well as, mesenchymal marker protein, vimentin. On the other hand, claudin-3, a tight junction protein depleted in CD44+/CD24- CSC enriched basal/mesenchymal breast tumors, was among the most down regulated genes in SK-β1-C1 (**Fig. S5**). Hierarchical clustering using the gene set (n = 1940) described above, showed that SK-β1-C1 clustered with HER-2 negative, claudin-low cell lines, MDA-MB-231 and MDA-MB-435. On the other hand, SKBR-3/EV, clustered with luminal cell lines, MCF-7 and ZR75-1 [12] (**Fig. 2c**). Western blotting for β1-integrin, vimentin and CK-18, revealed similar protein expression between claudin-low cell lines and colony cluster, SK-β1-C1 (**Fig. 2d**).

Based on the 1940 gene set, we found expression of several soluble factor genes was upregulated in SK-β1-C1 compared to SKBR-3/EV (**Table** **1**). Furthermore, secretion analysis showed elevated levels of IL-6 and TGF-β1, both known to induce EMT traits in an autocrine/paracrine manner [22], [30], in JIMT-1 and mesenchymal SKBR-3 colony clusters. Secretion of FGF-2, a factor involved in maintenance of SC pluripotency [31], was also increased in SK-β1-C1 and JIMT-1 cells (**Fig. S6**).

**Table 1:** Upregulation of soluble factor genes in SK-β1-C1 versus SKBR-3/EV cells.

Gene symbol	Gene Description	Fold-increase	*P*-value	GenBank
CSF1	colony stimulating factor 1 (macrophage)	29.6	0.016	NM_172210
CX3CL1	chemokine (C-X3-C motif) ligand 1	156.6	0.001	NM_002996
CXCL12	chemokine (C-X-C motif) ligand 12 (stromal cell-derived factor 1)	314.6	<0.001	NM_199168
FGF2	fibroblast growth factor 2 (basic)	352.0	0.012	NM_002006
FGF13	fibroblast growth factor 13	186.4	0.005	NM_004114
IGFBP3	insulin-like growth factor binding protein 3	26.2	0.018	NM_001013398
IGFBP6	insulin-like growth factor binding protein 6	2710.4	<0.001	NM_002178
IGFBP2	insulin-like growth factor binding protein 2, 36kDa	11.2	0.015	NM_000597
IL6	interleukin 6	48.9	0.011	NM_000600
IL7	interleukin 7	528.1	<0.001	NM_000880
IL15	interleukin 15	7.6	0.004	NM_172174
NRG1	neuregulin 1, transcript variant GGF	2331.1	<0.001	NM_013961
NRG1	neuregulin 1, transcript variant GGF2	964.0	<0.001	NM_013962
NRG1	neuregulin 1, transcript variant HRG-beta2	1417.5	< 0.001	NM_013957
TGFB1	transforming growth factor, beta 1	320.3	0.010	NM_000660
TGFB2	transforming growth factor, beta 2	7.2	0.017	NM_003238
TGFB2	transforming growth factor beta-2 precursor	6.7	0.007	ENST00000366930
TGFB3	transforming growth factor, beta 3	72.0	<0.001	NM_003239
TGFBI	transforming growth factor, beta-induced, 68kDa	61.7	0.016	NM_000358
VEGFC	vascular endothelial growth factor C	12.9	<0.001	NM_005429

### N-glycosylation of β1-integrin plays a functional role in the SKBR-3 EMT phenotype

**Figure 3:**
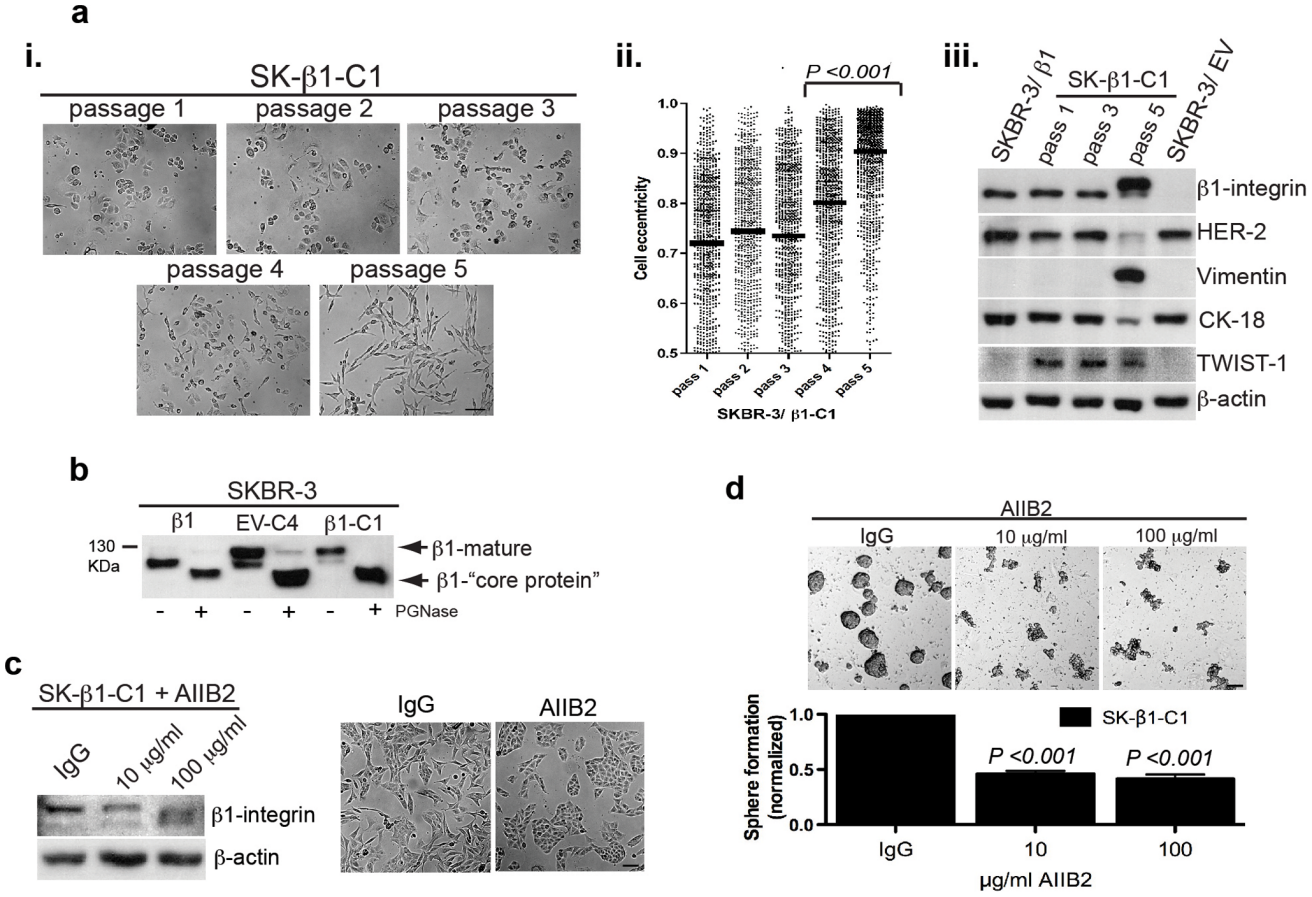
A functional role for N-glycosylated β1-integrin in the SKBR-3 EMT phenotype. [**a-i**] Images of sequential passages of SK-β1-C1 cells following colony isolation were digitized and analyzed using Cell Profiler© software. [**a-ii**] The accumulation of spindle shaped cells occurred rapidly between passage 4 and 5 (shape score: 0 = circle, 1 = line) (n = 850 cells analyzed/passage). [**a-iii**] Western blot analysis for EMT markers (vimentin, N-cadherin, TWIST-1) in SK-β1-C1 cells at the indicated passage. [**b**] Heavily N-glycosylated β1-integrin (β1-mature) is converted to β1-integrin “core protein” after lysate from indicated cell lines was treated with PGNase-F, an enzyme that removes N-glycan chains from core proteins. [**c**] Western blotting and images of SK-β1-C1 cells treated for 72h with β1-integrin inhibitory antibody, AIIB2, or IgG control. [**d**] Mammosphere formation in SK-β1-C1 cells following 10 days of treatment with AIIB2 or IgG control. Representative images at day 5 of treatment are shown.

In this study, SK-β1-C1 cells gained expression of a high molecular weight variant of β1-integrin following EMT. To investigate the relationship between observed β1-integrin variants and EMT, we returned to an early passage of colony cluster SK-β1-C1. Using cell shape as a phenotypic marker for EMT, we monitored sequential passaged of SK-β1-C1 cells. Notably, the quantity of spindle-shaped cells increased exponentially between passage 4 and 5, marking the point of EMT in SK-β1-C1 (*P*-value <0.001). Interestingly, by western blotting we confirmed that the molecular weight shift in β1-integrin coincided precisely with the expression of EMT markers and loss of HER-2 in SK-β1-C1 cells. EMT-inducer, TWIST-1, was expressed in passage 1 and 3 SK-β1-C1 cells, preceding the occurrence of EMT (**Fig. 3a**).

Alterations to the electrophoretic mobility of β1-integrin, typically corresponds to differences in N-glycosylation [27], [32]. In this regard, it appears that the 105 KDa ‘precursor’ form of β1-integrin becomes completely exchanged for the 130 KDa ‘mature’ form of the protein in passage 5 of SK-β1-C1 cells. To confirm if N-glycosylation accounts for the shift in electrophoretic mobility of β1-integrin, we used PGNase-F, an enzyme that hydrolyzes N-glycan chains from glycoproteins [33]. Lysates from SKBR-3/β1, SK-β1-C1, and SK-EV-C4 cells were treated with PGNase-F. Subsequent resolution of proteins by SDS-page confirmed increased N-glycan deposition on β1-integrin in mesenchymal colony clusters (**Fig. 3b**).

Interestingly, N-glycosylation patterns characterized by β1-6-branching have been associated with phenotype transitions and malignant behavior in cancer cells [34]. To determine if SKBR-3 mesenchymal colony clusters expressed increased β1-6 branch deposition, we used a fluorescently labeled plant lectin, L-PHA, with β1-6 binding specificity [34]. By confocal microscopy we found high intensity staining in SK-β1-C1 but not SKBR-3/β1 cells. Additionally, MGAT-5, a glycosyltransferase responsible for β1-6 branching of proteins, including β1-integrin, and associated with poor patient outcome, tumorigenesis and metastasis in BC [27], [35], [36], was upregulated (7.4 fold) in SK-β1-C1 cells (data not shown).

To evaluate a role for N-glycosylated β1-integrin in the basal/mesenchymal phenotype of SK-β1-C1, we used AIIB2, a β1-integrin antibody that may preferentially target the high molecular weight isoform of β1-integrin, and has been shown to revert the malignant phenotype in BC cells [37], [38]. Here, we found that treatment of SK-β1-C1 cultures with 10 and 100 μg/ml of AIIB2 decreased the N-glycosylated variant of β1-integrin. Remarkably, this was accompanied by a restoration of round epithelial morphology in SK-β1-C1 cells (**Fig. 3c**). To characterize other changes in EMT marker proteins caused by AIIB2 treatment, we performed additional western blotting of lysate treated with AIIB2 or IgG control. After protein expression was quantified and analyzed, our results showed that AIIB2 treatment of SK-β1-C1 cells induced an extensive down-regulation of β1-integrin and EGFR proteins, as well as, a modest down-regulation of phosphorylated (p)-FAK (Tyr397), vimentin, and TWIST-1 (**Fig. S7**).

Next, we treated mammosphere cultures from colony cluster SK-β1-C1 with AIIB2 or IgG control, and assessed sphere formation following 10 days of treatment. Notably, treatment with 10 or 100 μg/ml of AIIB2 resulted in a dramatic 54%, and 59% reduction in the number of mammospheres formed, respectively (*P*-value <0.001) (**Fig. 3d**). To determine if reduced mammosphere formation was the direct result of inhibited cell proliferation by AIIB2, we treated SK-β1-C1 cells with antibody. After 72 hours, both doses of AIIB2 treatment (10 and 100 μg/ml) resulted in a 19% reduction in cell proliferation in SK-β1-C1. These doses did not inhibit the proliferation of SKBR-3/EV cells (**Fig. S7**). This modest reduction in cell proliferation is unlikely to account for the greater than 50% reduction in mammosphere formation in SK-β1-C1 following AIIB2 treatment.

### EMT results in spontaneous resistance to HER-2 targeted therapies in SKBR-3 colony clusters

**Figure 4:**
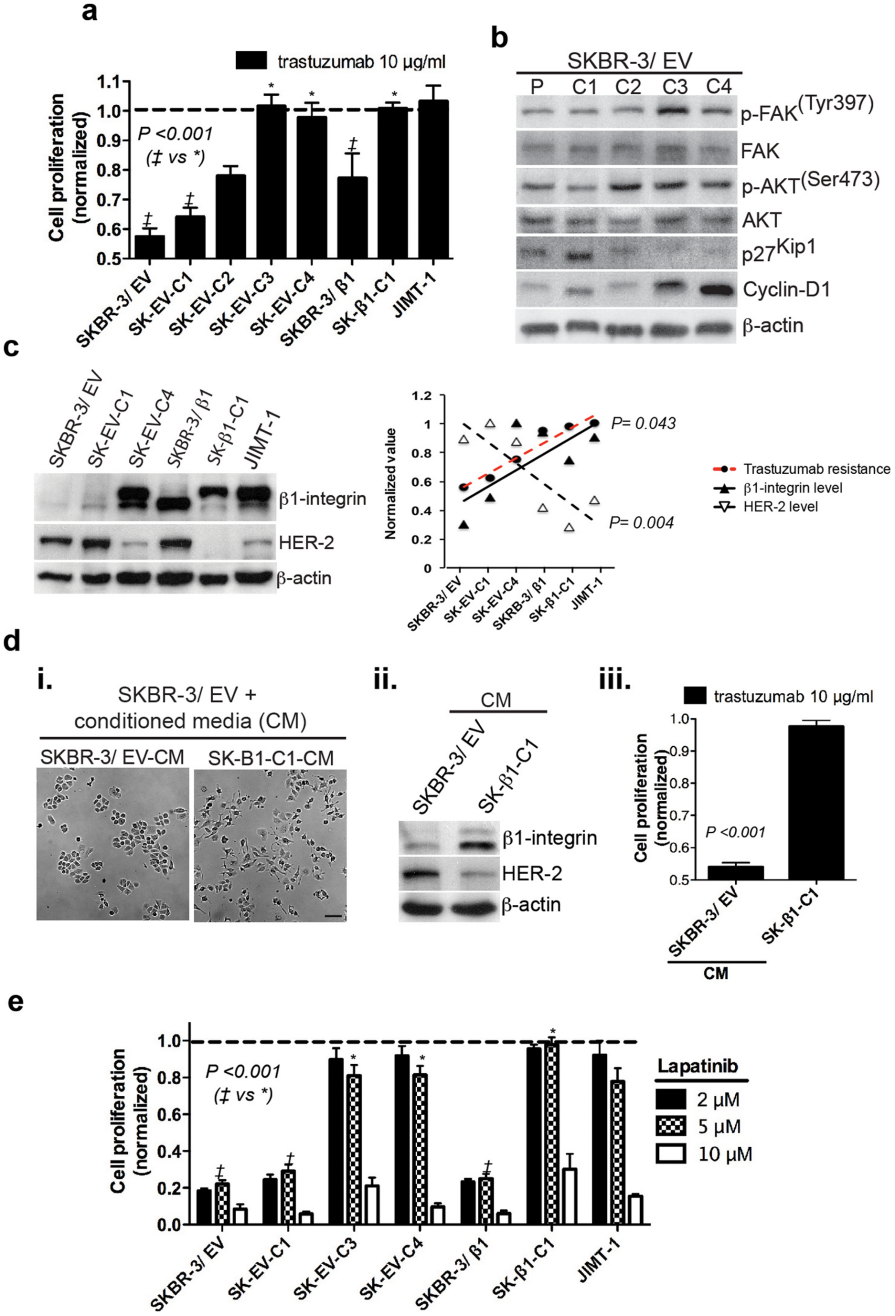
EMT in luminal HER-2+ SKBR-3 breast cancer cells mediates resistance to HER-2 targeted therapies. [**a**] Proliferation of SKBR-3 cell lines and JIMT-1 following 72 h treatment with trastuzumab or IgG control was assessed by XTT assay. Mesenchymal colony clusters (SK-EV-C3, EV-C4 and SK-β1-C1) are resistant to trastuzumab treatment, similar to JIMT-1 (dashed line). [**b**] Western blotting for trastuzumab resistance-associated proteins in SKBR-3/EV colony clusters (P = parental cell line). [**c**] Correlation of HER-2 and β1-integrin protein expression (left) with trastuzumab response (Fig. 4a) in luminal (SKBR-3/EV, SK-EV-C1, SKBR-3/β1 and mesenchymal (SK-EV-C4, SK- β1-C1, JIMT-1) cell lines. [**d**] SKBR-3/EV cells were cultured for 72 h in the presence of conditioned media (CM) obtained from SKBR-3/EV or SK-β1-C1. Images and western blotting showed increased spindle morphology (**d-i**), decreased levels of HER-2, and increased levels of β1-integrin (**d-ii**) in SK-β1-C1-CM treated cells. Cell proliferation following CM-exposure was assessed as previously described, and showed resistance to trastuzumab in SKBR-3/EV cells exposed to SK-β1-C1-CM (**d-iii**). [**e**] Proliferation of SKBR-3 colony clusters and JIMT-1 cells following 72 h treatment with lapatinib or vehicle control at indicated concentrations. Cell proliferation is expressed as a ratio to untreated control (normalized) [a], [e].

Finally, we used our model of spontaneous phenotype plasticity in luminal, HER-2+, trastuzumab sensitive SKBR-3 cells to investigate a role for EMT in resistance to trastuzumab. First, cell lines were treated with a common preclinical dose of trastuzumab, 10 μg/ml representing the ideal target serum level in patients [8]. Our results showed that, while luminal SKBR-3 cell lines remained sensitive to trastuzumab treatment, mesenchymal colony clusters were completely resistant (*P*-value <0.001) (**Fig. 4a**). Notably, all colony clusters isolated from JIMT-1 cells retained a high level of resistance to trastuzumab treatment (**Fig. S8**). Interestingly, western blotting of SKBR-3/EV colony clusters revealed altered expression of several TZR-associated proteins simultaneously in mesenchymal colony clusters. For instance, p-FAK (Tyr397), p-Akt (Ser473), and Cyclin-D1 were elevated in SK-EV-C3 and SK-EV-C4, while the level of p27 (KIP1) was decreased (**Fig. 4b**).

To determine if trastuzumab sensitivity was correlated with HER-2 and/or β1-integrin expression we quantified HER-2 and β1-integrin receptor levels based on protein expression. We used western blotting of HER-2 and β1-integrin expression for several luminal (SKBR-3/EV, SK-EV-C1, SKBR-3/β1) and mesenchymal (SK-EV-C4, SK-β1-C1, JIMT-1) cell lines. Normalized protein expression values were correlated to trastuzumab sensitivity as determined by XTT assay. We found a statistically significant positive correlation (r = 0.75, *P* = 0.043) between trastuzumab resistance and β1-integrin expression across cell lines analyzed. Additionally, we found a statistically significant negative correlation (r = −0.92, *P* = 0.004) between trastuzumab resistance and expression of HER-2. Next, we analyzed whether, or not, the degree of β1-integrin N-glycosylation was correlated with trastuzumab resistance. Our results showed a trend towards higher levels of N-glycosylation and trastuzumab resistance, but this did not reach statistical significance (*P* = 0.081) (**Fig. 4c**).

Next, we looked to determine if the mixture of growth factors and cytokines secreted by SK-β1-C1 cells could mediate resistance to trastuzumab in luminal SKBR-3/EV cells. To accomplish this, SKBR-3/EV cells were cultured in the presence of conditioned media by SK-β1-C1 or SKBR-3/EV (as control). Following 72 h, we observed spindle (mesenchymal) morphology in SKBR-3/EV cells exposed to SK-β1-C1-conditioned media. Furthermore, western blotting showed downregulated HER-2 levels and increased expression of both precursor and mature β1-integrin isoforms following treatment with SK-β1-C1 conditioned media. Remarkably, cells exposed to SK-β1-C1 conditioned media were also completely resistant to trastuzumab (*P*-value <0.001) (**Fig. 4d**).

Lapatinib, a small molecule inhibitor of EGFR and HER-2 signaling, was recently approved for use in trastuzumab refractory BC [39]. Interestingly, β1-integrin mediated signaling has been reported to play a role in lapatinib resistance in HER-2+ BC [38]. To determine if mesenchymal SKBR-3 colony clusters displayed resistance to lapatinib therapy, cells were treated with established doses of lapatinib [40] and proliferation was assessed by XTT assay. Following 72h of treatment, JIMT-1 cells, along with all SKBR-3 mesenchymal colony clusters, were resistant to 2 and 5 μm of lapatinib treatment. Luminal colony clusters, on the other hand, remained sensitive to all concentrations of lapatinib tested (*P*-value <0.001) (**Fig. 4e**). Together, these results suggest that mesenchymal colony clusters have shifted reliance away from the HER-signaling axis for growth and survival.

## Discussion

Despite ranking as the second leading cause of cancer-related death in women worldwide, significant advances are being made in the diagnosis and treatment of BC [41]. For example, the discovery of molecular biomarkers has helped standardize the classification and management of the disease. Two routine clinical biomarkers assessed in BC, include: ER and HER-2. ER, a marker of differentiated luminal epithelium, is expressed in ∼65% of all BCs. These tumors are generally well differentiated (low-grade) and less aggressive than ER-negative BCs. On the other hand, patients with basal or HER-2+ BC have poor survival outcomes. While HER-2 remains an important clinical biomarker, it fails to identify which HER-2+ patients are most likely to respond to treatment [42]. Consequently, high rates of drug resistance and cardiotoxictiy remain long standing problems associated with trastuzumab use [4].

Recent studies have found that BC cell lines, including those HER-2+, contain multiple CSC populations defined by distinct markers [43]. In this regard, CSC defined by a CD44+/CD24- phenotype are typically enriched in claudin-low cell lines that are HER-2 negative. However, emerging evidence suggests that these HER-2 negative CSC are not only present in HER-2+ cell lines, but may actually contribute to TZR [44]. In addition, the discordance rates for HER-2 expression between matching primary and metastatic tumors has been reported [45]. For example, in one study of 382 matched tumors, a discordance rate of 34% was observed. Notably, HER-2+ patients whose metastatic disease had converted to HER-2-negative on biopsy (23.6%) had a worse overall prognosis [46]. In another study, the HER-2 gene signature was diminished in residual tumor cells following treatment with conventional therapies, while the proportion of cells enriched for claudin-low, EMT/CSC gene expression, was increased [47]. Based on our evidence presented here, one potential explanation for these findings would be an EMT-mediated loss of HER-2 expression during tumor progression. Furthermore, our results highlight a potential role for the heavily N-glycosylated, mature isoform of β1-integrin in EMT and the claudin-low phenotype in BC cells.

Given the down-regulation of HER-2 in mesenchymal SKBR-3 colony clusters, the findings of trastuzumab resistance may seem largely predictable. However, multiple studies have demonstrated that trastuzumab treatment can be effective in BC with low or negative HER-2 expression. A study by Paik et al, showed no correlation between HER-2 gene amplification and response to adjuvant trastuzumab treatment. Furthermore, the authors found patients with normal levels of the HER-2 (so called “HER-2 negatives”) received benefit from adjuvant trastuzumab therapy [48]. A preclinical study by Diermeier et al, makes a similar conclusion that HER-2 expression status alone is not able to predict trastuzumab resistance in BC cell lines, including SKBR-3. Here the authors showed that sensitivity to trastuzumab is not solely based on HER-2 receptor status, but rather influenced by co-expression of alternative HER receptors and ligands. For instance, SKBR-3 cells co-express elevated levels of EFGR (HER1) compared to luminal, HER-2+ BT-474 cells. As consequence, when treated with trastuzumab, BT-474 cells are more likely to enter a quiescent state compared to SKBR-3. Additionally, treatment of both cell lines with neuregulin (NRG), a HER ligand, was able to completely compensate for trastuzumab mediated inhibition of cell proliferation [49].

To investigate changes in HER receptors and ligands between different SKBR-3 colony clusters, we used gene expression profiling. Interestingly, we found decreased levels of HER-2 and HER-3 in mesenchymal colony clusters compared to luminal cell lines (Fig. S3). EGFR levels remained consistent throughout all SKBR-3 cell lines, indicating that EGFR is unlikely to account for the trastuzumab resistant phenotype in mesenchymal colony clusters. Notably, the levels of NRG were significantly upregulated in mesenchymal but not luminal colony clusters (Table 1). Together with modulation of p-AKT, p-FAK, Cyclin-D1, and p27 (KIP1) (Fig. 4b), up-regulated NRG levels represent an additional regulator of trastuzumab response that is modulated by EMT in SKBR-3 cells.

According to the “intrinsic” model for cell culture heterogeneity, phenotype divergence occurs inherently as cell-fate determinants are asymmetrically segregated during cell division. In comparison, the “extrinsic” model proclaims that changes in the local environment influence cell differentiation [50]. While our SKBR-3 model provides support for the latter, the conversion of SKBR-3 cells from a luminal to a claudin-low phenotype should be recognized in light of an important caveat. First, the fact that SKBR-3 cells are negative for two functionally important luminal markers, ER and E-Cadherin, may have facilitated their shift in phenotype. Second, a comprehensive survey of 51 BC cell lines showed the claudin-low phenotype to be enriched in 27% of cell lines. This is compared to less than 10% of breast tumors enriched for the same basal/mesenchymal traits (claudin-low) [3], [12].

These figures suggest that cell culture conditions may provide a more favorable environment for propagation of the claudin-low phenotype. Future studies using additional cell lines, including: trastuzumab sensitive, HER-2+ BT-474 cells (ER and E-cadherin positive), are therefore warranted.

In conclusion, our data highlight the role of EMT in TZR in luminal HER-2+ BC. We demonstrated the functional significance of a heavily N-glycosylated variant of β1-integrin in the EMT phenotype. Furthermore, while variant N-glycosylation is an established hallmark of cancer, specific N-glycosylation targets remain a relatively untapped class of biomarkers and therapeutic targets [51]. Accordingly, most previous studies of β1-integrin have focused primary on intracellular signaling pathways and cell-matrix interactions. Our findings presented here represent a novel link between EMT, N-glycosylated β1-integrin and the therapeutic consequences of cell plasticity in luminal HER-2+ BC cells.

**The authors of this publication declare that all experiments described herein comply with the law of the country in which they were performed.**

## Supporting Information

Figure S1 **Isolation of colony clusters from HER-2+ SKBR-3 breast cancer cells.** [**Left**] Morphological heterogeneity is apparent among clones from HER-2 positive luminal cell line, SKBR-3, following 10–12 days of growth under standard conditions. Round, epithelial “grape-like” colonies (1) and dispersed, mesenchymal “spindle” colonies (2) were observed. [**Right**] Cells were plated at a density of 10,000 cells/ml (10 ml) in a 100 mm culture dish and allowed to grow for 10–12 days under standard conditions. Using multiple cloning rings (10 mm), distinct colony clusters (C1–4) were isolated randomly from a single dish following day 10–12 in culture. Crystal violet staining was used here to illustrate cell density and the cloning technique.
(TIF)

Figure S2 **Consecutive mammosphere formation in SK-β1-C1 cells.** Mammosphere forming ability of SK-β1-C1 cells was assessed for 5 consecutive passages. A representative image from third passage mammospheres following 10 days in culture is shown.
(TIF)

Figure S3 **Gene expression differences for HER family receptors and ligands.** Relative gene expression differences for HER receptors and ligands, between SKBR-3/EV, SKBR-3/β1, mesenchymal colony clusters, SK-EV-4 and SK-β1-C1.
(TIF)

Figure S4 **Protein expression differences for key EMT/CSC-markers.** Western blotting [**Right**] and FACs analysis [**Left**] were used to validate gene expression differences described in Figure 2.
(TIF)

Figure S5 **Top upregulated and downregulated genes in SKBR-3 colony clusters.** Top ten upregulated (black bars) and downregulated (white bars) genes based on the 1940 gene set in SK-β1-C1 compared to SKBR-3/EV. Collagen-1A2 (COL1A2), lamanin-alpha-4 (LAMA4), and vimentin (VIM), were among the most upregulated genes. Claudin-3 (CLDN3) was among the most down regulated genes (boxes). Expression differences are plotted according to statistical significance as −log (*P*).
(TIF)

Figure S6 **Secretion of EMT-related soluble factors in SKBR-3 colony clusters.** Secretion of EMT-related soluble factors was assessed by ELISA assays using the supernatant from confluent cultures (72h) of indicated cell lines. Values displayed are normalized to cell number.
(TIF)

Figure S7 **Western blot analysis of SK-β1-C1 cells treated with AIIB2.** Western blotting for EMT/mesenchymal associated markers in SK-β1-C1 cells treated for 72 h with 100 μg/ml β1-integrin inhibitory antibody, AIIB2, or IgG control [**Left**]. Graphical representation of protein expression data [**Middle**]. The effect of AIIB2 treatment on cell proliferation in luminal and mesenchymal breast cancer cell lines. Cells were grown under standard conditions and proliferation was determined by counting cells after 72 h [**Right**].
(TIF)

Figure S8 **Response of JIMT-1 colony clusters to treatment with trastuzumab.** JIMT-1 cell lines were treated with trastuzumab or control IgG1 for 72 h and cell proliferation was assessed by XTT assay. All JIMT-1 clones remained resistant to trastuzumab treatment (dashed line).
(TIF)

Methods S1 **Supplemental materials and methods.**
(DOC)
