# Supplementary figures and images for: Spontaneous Epithelial-Mesenchymal Transition and Resistance to HER-2-Targeted Therapies in HER-2-Positive Luminal Breast Cancer
Source: PLoS One. 2013 Aug 26;8(8):e71987. doi: 10.1371/journal.pone.0071987 (PMC3753362; doi:10.1371/journal.pone.0071987)

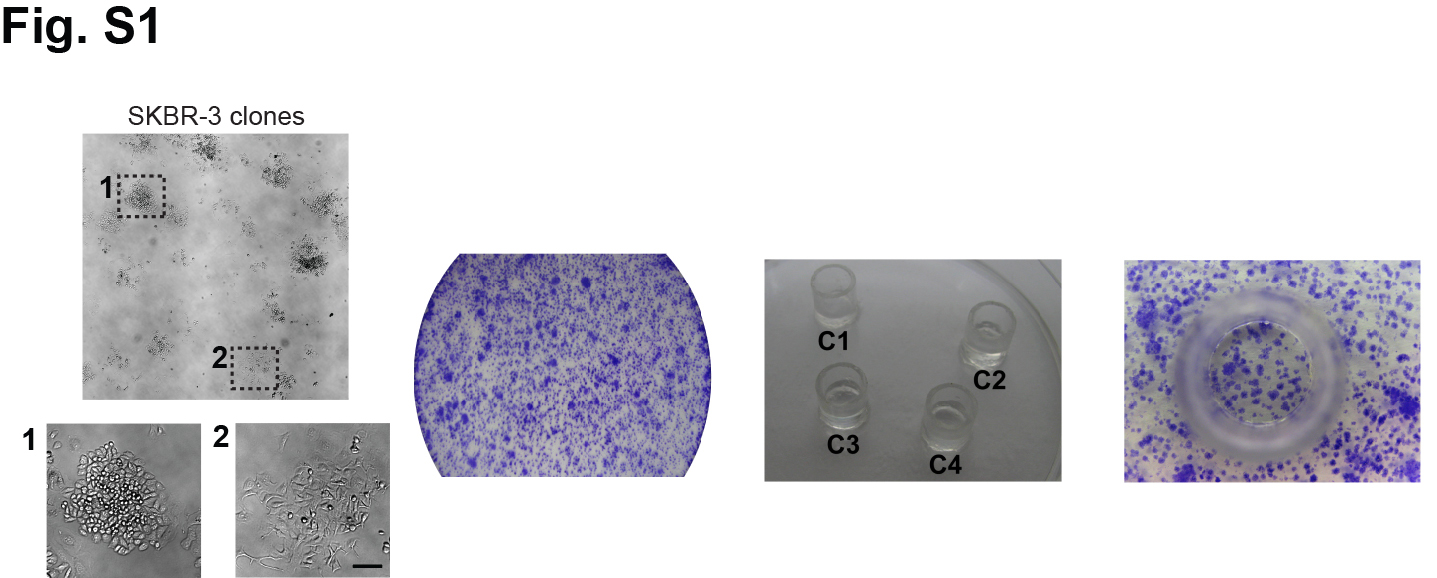

Supplement: Figure S1 — Isolation of colony clusters from HER-2+ SKBR-3 breast cancer cells. [Left] Morphological heterogeneity is apparent among clones from HER-2 positive luminal cell line, SKBR-3, following 10–12 days of growth under standard conditions. Round, epithelial “grape-like” colonies (1) and dispersed, mesenchymal “spindle” colonies (2) were observed. [Right] Cells were plated at a density of 10,000 cells/ml (10 ml) in a 100 mm culture dish and allowed to grow for 10–12 days under standard conditions. Using multiple cloning rings (10 mm), distinct colony clusters (C1–4) were isolated randomly from a single dish following day 10–12 in culture. Crystal violet staining was used here to illustrate cell density and the cloning technique. (TIF) [file pone.0071987.s001.tif]

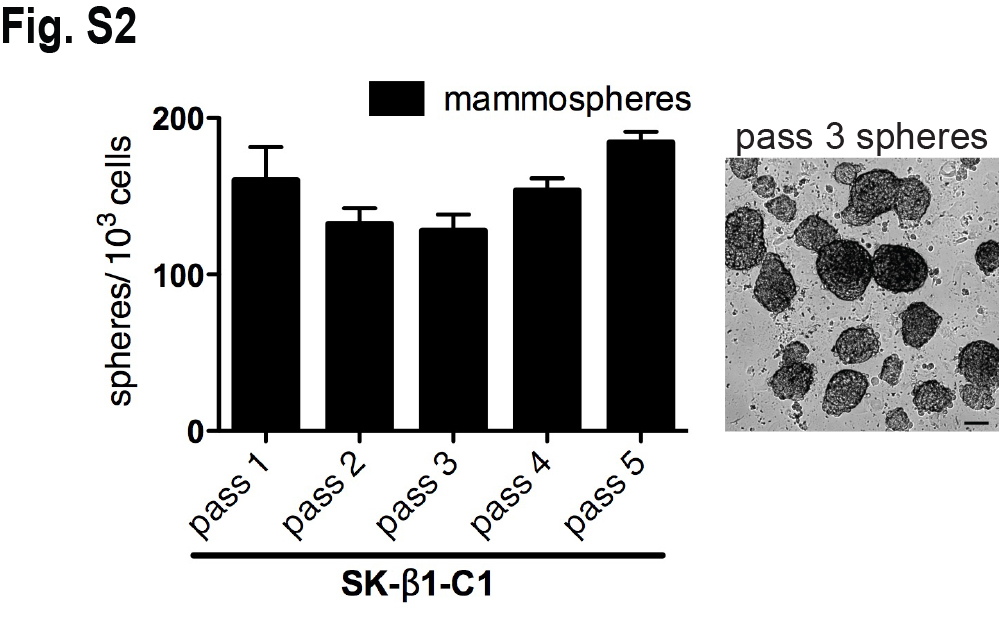

Supplement: Figure S2 — Consecutive mammosphere formation in SK-β1-C1 cells. Mammosphere forming ability of SK-β1-C1 cells was assessed for 5 consecutive passages. A representative image from third passage mammospheres following 10 days in culture is shown. (TIF) [file pone.0071987.s002.tif]

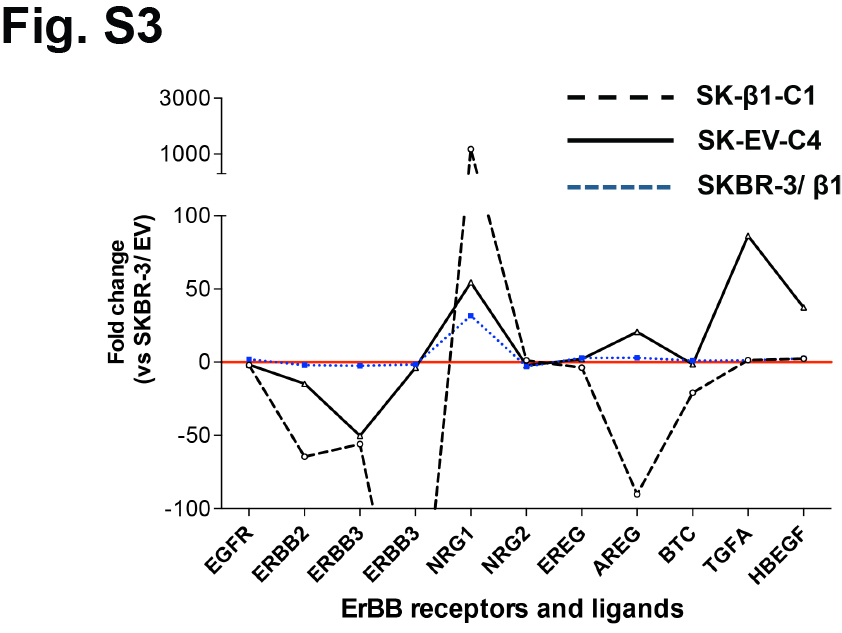

Supplement: Figure S3 — Gene expression differences for HER family receptors and ligands. Relative gene expression differences for HER receptors and ligands, between SKBR-3/EV, SKBR-3/β1, mesenchymal colony clusters, SK-EV-4 and SK-β1-C1. (TIF) [file pone.0071987.s003.tif]

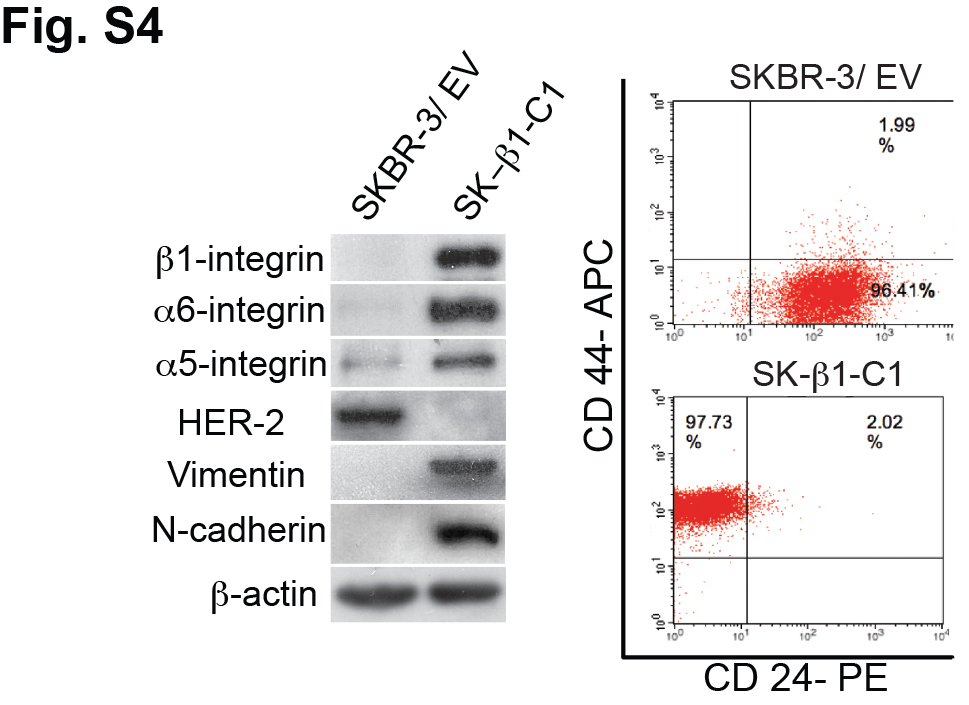

Supplement: Figure S4 — Protein expression differences for key EMT/CSC-markers. Western blotting [Right] and FACs analysis [Left] were used to validate gene expression differences described in Figure 2. (TIF) [file pone.0071987.s004.tif]

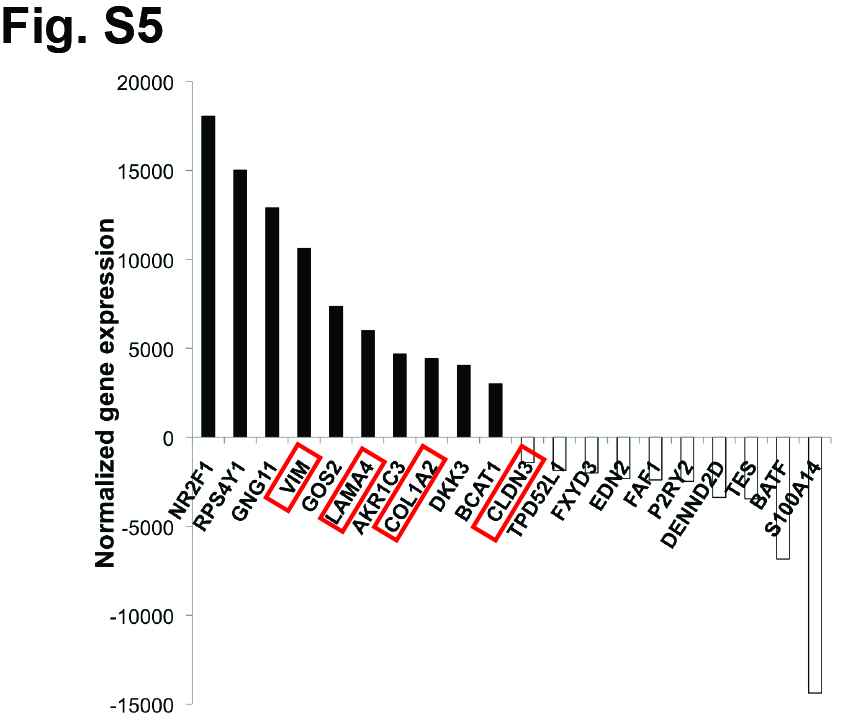

Supplement: Figure S5 — Top upregulated and downregulated genes in SKBR-3 colony clusters. Top ten upregulated (black bars) and downregulated (white bars) genes based on the 1940 gene set in SK-β1-C1 compared to SKBR-3/EV. Collagen-1A2 (COL1A2), lamanin-alpha-4 (LAMA4), and vimentin (VIM), were among the most upregulated genes. Claudin-3 (CLDN3) was among the most down regulated genes (boxes). Expression differences are plotted according to statistical significance as −log (P). (TIF) [file pone.0071987.s005.tif]

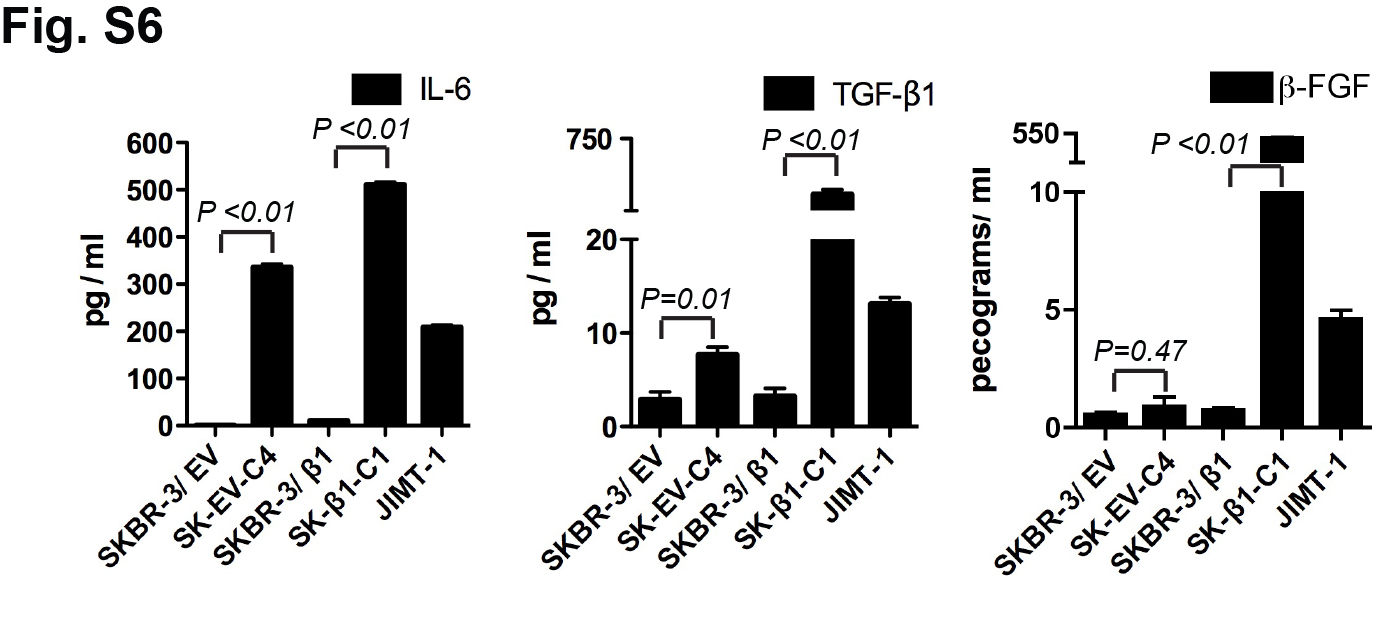

Supplement: Figure S6 — Secretion of EMT-related soluble factors in SKBR-3 colony clusters. Secretion of EMT-related soluble factors was assessed by ELISA assays using the supernatant from confluent cultures (72h) of indicated cell lines. Values displayed are normalized to cell number. (TIF) [file pone.0071987.s006.tif]

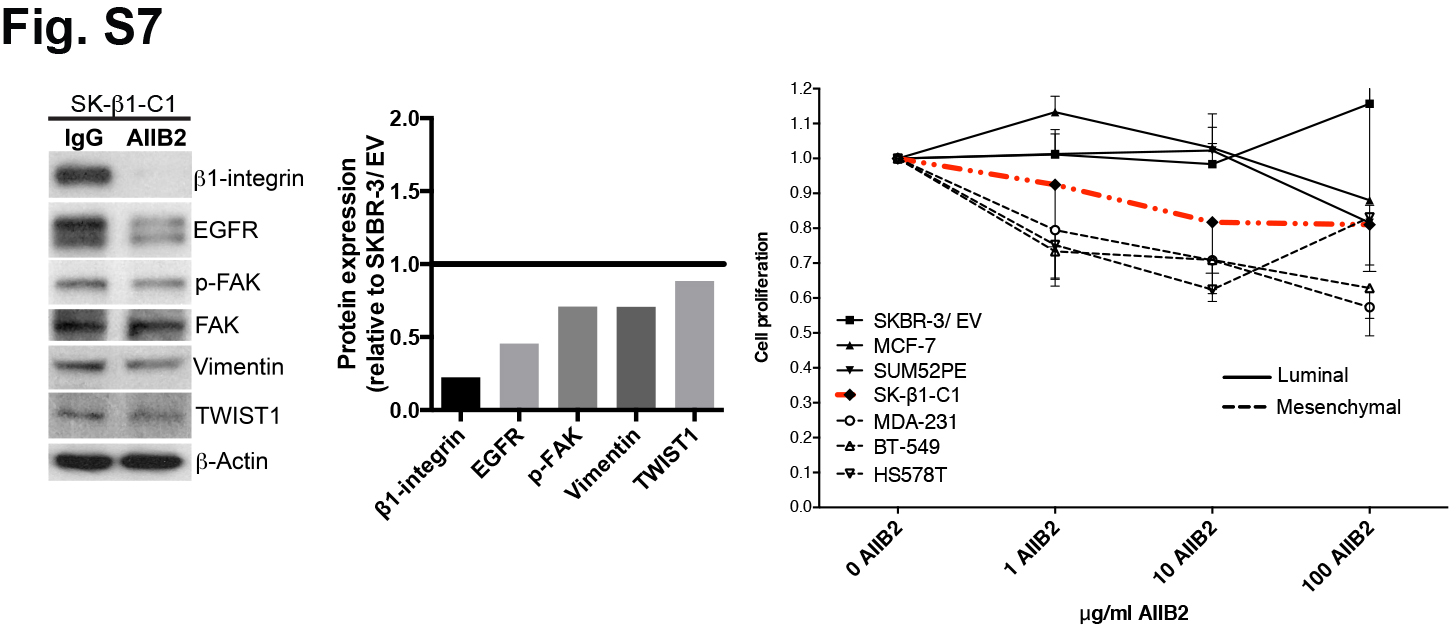

Supplement: Figure S7 — Western blot analysis of SK-β1-C1 cells treated with AIIB2. Western blotting for EMT/mesenchymal associated markers in SK-β1-C1 cells treated for 72 h with 100 μg/ml β1-integrin inhibitory antibody, AIIB2, or IgG control [Left]. Graphical representation of protein expression data [Middle]. The effect of AIIB2 treatment on cell proliferation in luminal and mesenchymal breast cancer cell lines. Cells were grown under standard conditions and proliferation was determined by counting cells after 72 h [Right]. (TIF) [file pone.0071987.s007.tif]

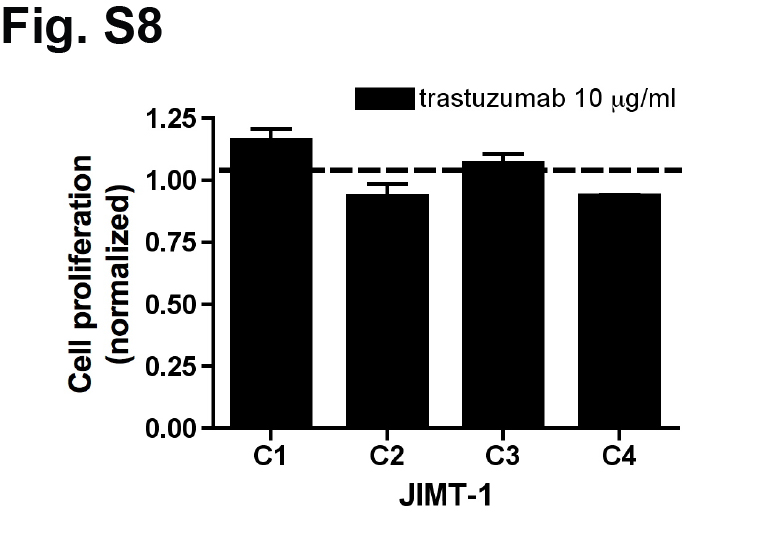

Supplement: Figure S8 — Response of JIMT-1 colony clusters to treatment with trastuzumab. JIMT-1 cell lines were treated with trastuzumab or control IgG1 for 72 h and cell proliferation was assessed by XTT assay. All JIMT-1 clones remained resistant to trastuzumab treatment (dashed line). (TIF) [file pone.0071987.s008.tif]
