# Supplementary material for: Spontaneous Epithelial-Mesenchymal Transition and Resistance to HER-2-Targeted Therapies in HER-2-Positive Luminal Breast Cancer
Source: PLoS One. 2013 Aug 26;8(8):e71987. doi: 10.1371/journal.pone.0071987 (PMC3753362; doi:10.1371/journal.pone.0071987)
Supplement: Methods S1 — Supplemental materials and methods. (DOC) [file pone.0071987.s009.doc]

**Supplemental materials and methods:**

**Cytokine Secretion:** Enzyme-linked immunosorbent assays (DuoSet ELISA, R&D systems) were used to determine the secretion of IL-6, TGF-β1 and β-FGF. Equal numbers of cells from each cell line (100,000 cells/well in 24 well plate) were grown overnight in 1 ml of standard (IL-6) or serum starved media (FGF-β and TGF-β1). The next day, media was collected and spun at 500g for 3min to remove debris and dead cells. Assays were completed as per manufacturer’s instructions, and concentrations shown are based on an internal standard curve generated for each experiment.

**FACs analysis:** Cells were labeled with anti-human CD24-PE (cloneSN3, AbCam) and anti-human CD44-Allophycocyanin (clone G44-26, BD Pharmingen) in 1× PBS/ 0.1% BSA/ 0.1% sodium azide (Sigma) at 4°C for 30minutes. Labeled cells were analyzed by flow cytometry.

**Mammosphere assay:** For continuous mammosphere culture, spheres were collected using an 80uM filter, washed with PBS and dissociated into single cells with Accutase Cell Dissociation Reagent (StemPro, Invitrogen). Mammosphere assay was carried out as previously described.

**AIIB2 production and purification:**

The hybridoma for β1-integrin function-blocking antibody, clone AIIB2 (Developmental Studies Hybridoma Bank, University of Iowa) was grown in Iscove DMEM containing 20% FBS and 50 µg/ml gentamicine. Cells positive for β1-integrin antigen were sorted by fluorescence activated cell sorting (FACS) and grown in Hyperflask culture vessels (Corning) with 10 gas permeable layers. Supernatant was purified using Protein L affinity chromatography column (Sigma). The column was washed with PBS until the absorbance at 280nm approached baseline. IgG was eluted as 1 ml fractions with glycine-HCL, pH2.8, neutralized with 1M Tris pH 9.0. IgG fractions were pooled together and dialyzed overnight against PBS. **AIIB2 screening by indirect ELISA:** Briefly, flat bottom 96 well plates (Nunc-ImmunoMaxisorb) were coated with purified β1-integrin antigen (5µg/ml) overnight at 4°C. Wells were washed 3 x with PBS and plate was blocked with 3% bovine serum albumin (2 h at room temperature (RT)). The plate was washed and 100 µl of purified AIIB2 was added for 2 hr at RT. Following three washes with PBS, bound antibodies were detected using Anti-Rat IgG conjugated with horseradish peroxidase (Sigma) for 1 hr at RT. After final washing, chemiluminescent substrate (KPL) was added to each well for 15 min at RT and the color was measured at 650nm using a microplate reader (Molecular Devices Corp, California).
